# Ovarian Metastasis from Lung Cancer: A Rare Entity

**DOI:** 10.1155/2013/378438

**Published:** 2013-03-24

**Authors:** Huseyin Cengiz, Şükrü Yıldız, Cihan Kaya, Erdinç Şenyürek, Murat Ekin, Levent Yasar

**Affiliations:** Bakirkoy Dr. Sadi Konuk Teaching and Research Hospital, Tevfik Sağlam Street, No. 11, Zuhuratbaba, Bakirkoy, 34147 Istanbul, Turkey

## Abstract

This paper describes a case of ovarian metastasis from lung carcinoma along with its diagnostic challenges, clinical management, and review of the literature. A 49-year-old woman was admitted to our emergency department with complaints of abdominal pain and vomiting. A laparoscopic appendectomy was performed due to acute appendicitis, and a unilateral oophorectomy (left side) via laparoscopy was performed due to the detection of an ovarian mass. Immunohistochemical staining of the ovarian mass revealed that it was reactive to cytokeratin-7 (CK-7) but negative for CK-20. The immunohistochemical and pathological features of the tumor indicated an ovarian metastasis of non-small-cell lung cancer. The patient underwent chemotherapy and was followed up by the oncology department. Her postoperative regular followup of 6 months showed that her condition was stable with no recurrence. The management of female patients with acute abdominal pain and pelvic masses should consist of a multidisciplinary approach to include the diagnosis of any distant organ metastasis.

## 1. Introduction

The majority of adnexal masses are functional cysts, endometriomas, teratomas, cystadenomas, leiomyomas, and malignant tumors [1]. However, the leading malignancy of the adnexa is ovarian carcinoma, which can be either a primary ovarian neoplasm or a metastasis from other sites of the body. In case of the latter, the metastasis originates mostly from tumors of the uterus, peritoneum, gall bladder, adrenal glands, and fallopian tubes. Approximately 4%–8% of ovarian malignancies are metastases from gastrointestinal tract or breast tumors [2]. Tumors may spread to the ovary via a blood-borne or lymphatic route, transperitoneally, or by direct extension. Differentiating between primary ovarian carcinoma and metastatic carcinoma is very important because the treatment regimen and prognosis are markedly different for each [3]. A multidisciplinary approach that includes clinical, serological, pathological, and radiological assessments is necessary in these cases. With a marked increase in its incidence, bronchogenic carcinoma is a leading cause of tumor-related deaths in many developed countries. Approximately 40% patients with bronchogenic carcinoma have metastasis on diagnosis [4]. The literature, however, contains only a few cases of bronchogenic carcinoma diagnosed after the detection of an ovarian metastasis [5].

The present paper is the first to describe an incidental diagnosis of bronchoalveolar carcinoma in a patient with adnexal mass presenting with acute abdominal pain.

## 2. Case Report

**Figure 1:**
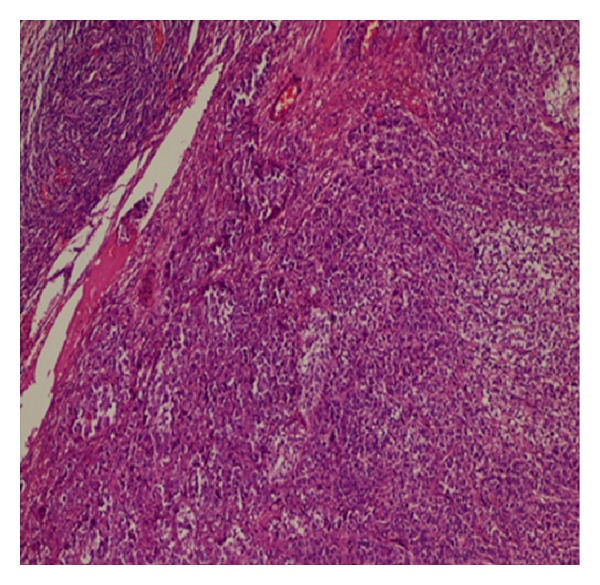
Photomicrograph showing stroma of ovaries and the rest of the staining lesion showing carcinoma (hematoxylin and eosin, ×100).

**Figure 2:**
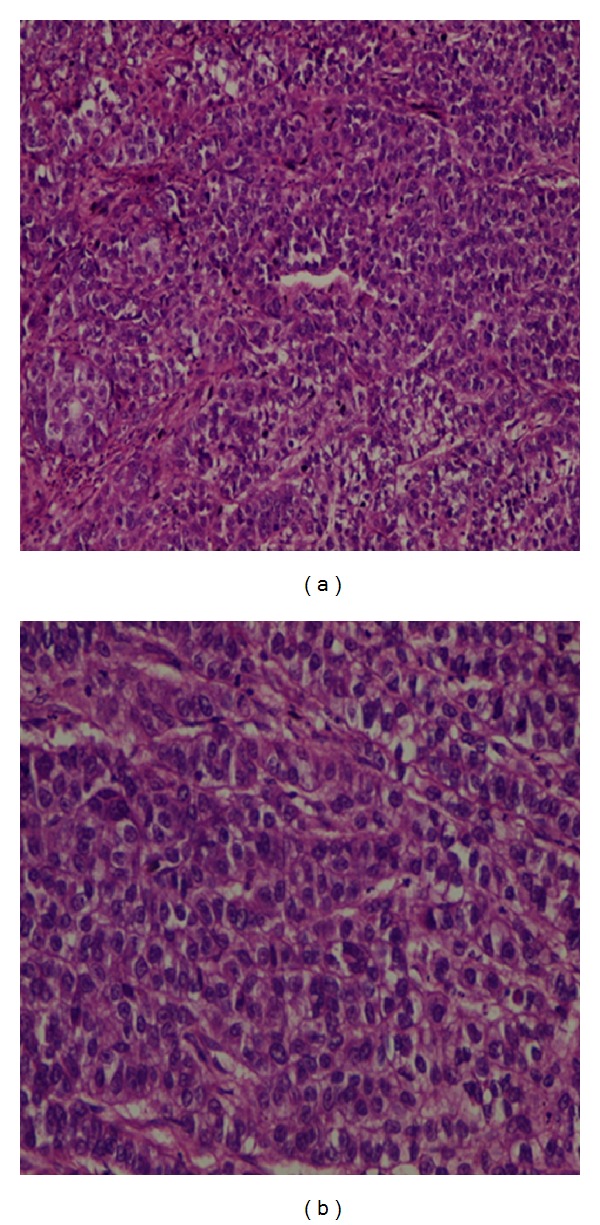
Histopathological findings. Lung adenocarcinoma metastasis. (a) Hematoxylin and eosin, ×200; (b) hematoxylin and Eosin, ×400.

**Figure 3:**
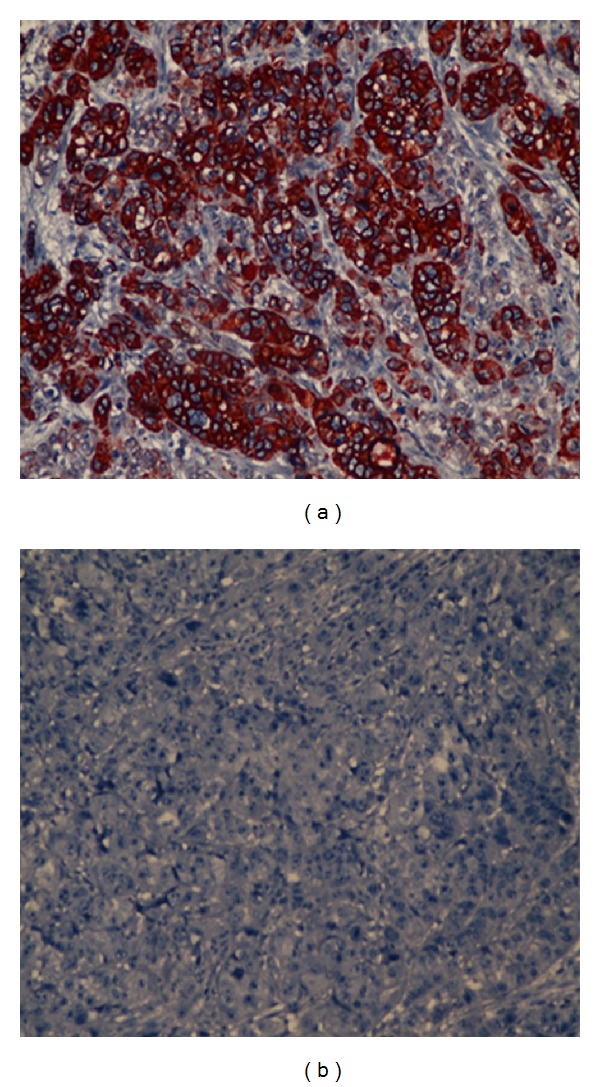
Immunohistochemical stainings. (a) Cytokeratin-7 staining reveals strong reactivity on the lung carcinoma. (b) Adenocarcinoma immunohistochemically negative for cytokeratin-20.

**Figure 4:**
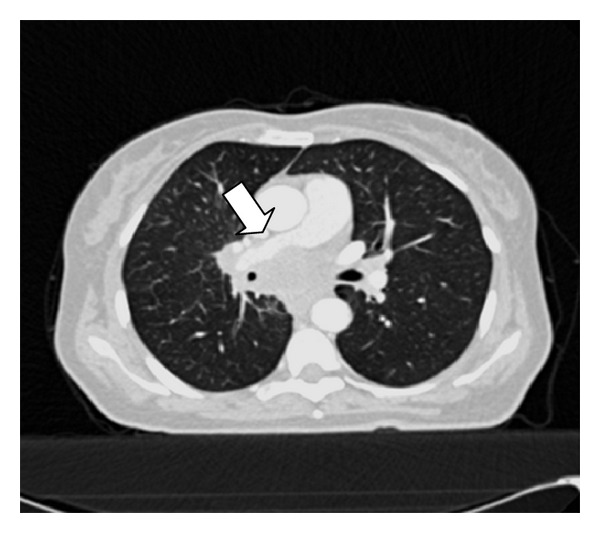
Axial CT image showing well-defined mass lesion causing mild compression of pulmonary artery (arrow).

A 49-year-old woman was admitted to our emergency department with complaints of abdominal pain and vomiting. She had no vaginal bleeding or discharge, diarrhea, fever, or constipation, and her medical history was unremarkable. Physical examination revealed tenderness and guarding at the right lower abdominal quadrant. An abdominal ultrasonography revealed a multiloculated 3 × 4 cm complex cyst in the left ovary and was positive for acute appendicitis. Subsequently, a laparoscopic appendectomy was performed by general surgeons; the appendix was found to be acutely inflamed and nonperforated. After an intraoperative gynecological consultation, a laparoscopic left oophorectomy was performed, and the tumor mass was removed using an endobag. To identify any possible ovarian malignancy, tumor markers were evaluated, and the results were as follows: CEA, 15.1 ng/mL (normal range, 0–3 ng/mL); CA 125, 49.5 ng/mL (0–35 ng/mL); CA15-3, 42.1 ng/mL (0–31 ng/mL); and CA 19-9, 76 U/mL (0–35 U/mL). On the basis of the pathology report, the tumor mass was diagnosed to be a well-differentiated adenocarcinoma of the left ovary (Figures 1 and 2). Immunohistochemical staining revealed that the tumor cells were reactive to cytokeratin-7 (CK-7, Figure 3(a)), but were negative for CK-20 (Figure 3(b)). The immunohistochemical and pathological features of the tumor were concordant with those of non-small-cell lung cancer with ovarian metastasis. Chest radiography revealed no pathology before or after the surgery. Computed tomography scans of the chest revealed a 6 cm mass in the mediastinum with mediastinal lymph node involvement and compression of the pulmonary artery (Figure 4). The patient underwent chemotherapy and was followed up by the oncology department. Her condition was stable during a regular followup of 6 months.

## 3. Discussion

Malignancy is one of the most common causes of death, and most patients die of complications of the metastatic disease. Bronchogenic carcinoma is the leading cause of tumor-related deaths in developed countries [3]. Adenocarcinoma accounts for approximately one-third of the lung carcinomas that metastasize to the ovary. Metastatic lung adenocarcinomas mimic ovarian surface epithelial-stromal tumors, such as the endometrioid, serous, clear cell, and mucinous type [6]. The ovary is an uncommon location for metastasis of lung cancer, and few such cases have been reported in the literature [3, 7–9]. Young and Scully reported 7 cases of ovarian metastasis of lung cancer [10]. In that report, ovarian tumors were detected before (*n* = 3), synchronously with (*n* = 3), or less than 1 year after (*n* = 1) the diagnosis of primary lung cancer. Young and Scully also pointed out that the clinical characteristics and pathological features of the disease may be useful in the diagnosis of ovarian metastasis from lung cancer. Irving and Young [6] reported 32 cases of lung carcinoma metastatic to the ovary. A history of prior lung carcinoma was documented in 17 (53%) of the 32 patients. In 10 (31%) patients, the lung and ovarian tumors occurred synchronously, and in 5 (16%), the ovarian tumor was detected up to 26 months before lung cancer diagnosis. Mazur et al. analyzed 325 cases of metastases to the female genital tract and reported only 1 case of synchronously diagnosed ovarian metastasis of lung cancer [11]. 

In our case, the patient was 49 years old and was admitted to our emergency department with acute abdominal pain. None of her symptoms reflected any pulmonary disease. In addition, accurate preoperative evaluation could not be performed due to the emergency conditions. However, laparoscopic surgery and incidental diagnosis of the primary disease sets this case apart from the rest. 

In recent years, immunohistochemistry studies with CK-7 and CK-20 staining have been widely used to differentiate between primary and secondary ovarian malignancies. Yeh et al. were the first to point out the importance of immunohistochemical staining [8]. In the present case, the well-differentiated adenocarcinoma of the left ovary was first detected pathologically. Later, immunohistochemical staining was used to detect the primary source of the cancer. 

## 4. Conclusion

The management of abdominal masses should include a multidisciplinary approach. Female patients likely to have a pelvic mass should be referred to a gynecologist before deciding on any abdominal surgeries. Radiological findings correlating with those of pathological and immunohistochemical staining are required for the diagnosis of metastatic ovarian tumors.
